# Psychometric properties of patient-reported outcome measures for symptom assessment in patients with cancer receiving immunotherapy: A systematic review following the COSMIN 2.0 guidelines

**DOI:** 10.1016/j.apjon.2025.100774

**Published:** 2025-08-19

**Authors:** Defa Zhang, Yali Wang, Qian Wang, Huiqing Mao, Miaomiao Zhang, Ping Xu, Shuo Guo, Rong Yan

**Affiliations:** a School of Nursing, Shandong First Medical University(Shandong Academy of Medical Sciences), Jinan, China; b Department of Respiratory Internal Medicine Ward 1, Shandong Cancer Hospital and Institute, Shandong First Medical University and Shandong Academy of Medical Sciences, Jinan, China; c Department of Ovarian Tumor Ward, Shandong Cancer Hospital and Institute, Shandong First Medical University and Shandong Academy of Medical Sciences, Jinan, China; d Department of Respiratory Internal Medicine Ward 2, Shandong Cancer Hospital and Institute, Shandong First Medical University and Shandong Academy of Medical Sciences, Jinan, China; e Department of Intensive Care Unit, Shandong Cancer Hospital and Institute, Shandong First Medical University and Shandong Academy of Medical Sciences, Jinan, China; f Department of Prevention Management, Shandong Cancer Hospital and Institute, Shandong First Medical University and Shandong Academy of Medical Sciences, Jinan, China

**Keywords:** Psychometric property, Patient-reported outcome measures, Neoplasms, Immunotherapy, COSMIN

## Abstract

**Objective:**

To identify and evaluate the methodological quality and psychometric properties of Patient-reported outcome measures (PROMs) for symptom assessment in patients with cancer undergoing immunotherapy.

**Methods:**

A systematic search was performed in PubMed, Scopus, Cochrane Library, Web of Science, Embase, CINAHL, CNKI, WanFang, Vip, and SinoMed from their inception to February 10, 2025. Eligibility criteria required studies to focus on the development or validation of a PROM for symptom assessment in adult patients with cancer undergoing immunotherapy, and to report on at least one psychometric property. The methodological quality of the included studies and the psychometric properties of the corresponding instruments were evaluated using the COSMIN 2.0 guidelines.We synthesized the evidence pertaining to each measurement property from all included studies on the same PROM.The evidence quality was evaluated based on an adapted Grading of Recommendations Assessment, Development and Evaluation system.

**Results:**

The final analysis included eight studies that reported on six PROMs. Because there was insufficient high-quality evidence, particularly for cross-cultural validity/measurement invariance and measurement error, no instrument could be recommended for use. However, the Functional Assessment of Cancer Therapy-Immune Checkpoint Modulator (FACT-ICM) demonstrated the most potential, with high-quality evidence supporting its content validity, structural validity, and internal consistency. Notably, the primary goal of this instrument is to assess the overall symptom burden.

**Conclusions:**

There is a notable lack of fully validated, high-quality instruments for measuring the symptoms themselves in patients undergoing immunotherapy. Although the FACT-ICM is the most promising tool for assessing the overall symptom burden, it still requires further validation. Therefore, future research, depending on the specific objective, should focus on two key areas: more comprehensive validation of the FACT-ICM, and the validation or development of instruments specifically designed to quantify the symptoms themselves.

**Systematic review registration:**

PROSPERO (CRD420250655464).

## Introduction

Cancer represents a primary global public health challenge in the 21st century. The International Agency for Research on Cancer reported that in 2022, there were nearly 20 million new cancer cases and 9.7 million deaths worldwide.^1^ This stark reality poses a formidable challenge to health care systems globally and demands continuous innovation in cancer prevention and treatment strategies.^2^ Against this backdrop, novel cancer immunotherapies, including immune checkpoint inhibitors (ICIs) and chimeric antigen receptor (CAR) T-cell therapy, have produced substantial gains in survival for patients with a range of malignancies.^3^^,^^4^ However, this revolution, which has profoundly reshaped the landscape of oncology, also presents unique and substantial challenges for cancer nursing practice.^5^^,^^6^

Unlike conventional treatments, immunotherapy induces a unique profile of toxicities, known as immune-related adverse events (irAEs), that can affect various organ systems such as the skin, gastrointestinal tract, and musculoskeletal system.^7^, ^8^, ^9^ These symptoms not only impair patients' quality of life but can, in severe cases, lead to treatment interruption or become life-threatening.^6^^,^^10^ Consequently, the precise and timely monitoring and assessment of these symptoms have become critical for ensuring patient safety and treatment continuity, representing a core metric of high-quality, contemporary oncology nursing.

Although symptom monitoring has traditionally been performed by health care professionals, a substantial body of research indicates that the actual intensity of patient symptoms is frequently underrated by health care professionals.^11^, ^12^, ^13^ This can lead to delayed symptom recognition, inadequate intervention, and compromised treatment adherence and outcomes. Therefore, a major international trend in both general and oncology nursing practice is the increasing use of patient-reported outcomes (PROs) for symptom assessment.^14^ By capturing the severity and fluctuation of subjective symptoms directly from the patient, Patient-Reported Outcome Measures (PROMs) yield a more complete, genuine, and dynamic form of clinical information that is essential for personalizing patient-centered care.^15^

The foundation of robust and reliable assessment lies in the use of accurate and reproducible instruments.^16^^,^^17^ For patients undergoing cancer immunotherapy, several specific symptom assessment tools are available, including the MD Anderson Symptom Inventory for early-phase (immunotherapy) trials (MDASI-Immunotherapy EPT),^18^^,^^19^ the MD Anderson Symptom Inventory for chimeric antigen receptor T-cell therapy (MDASI-CAR),^20^ and the Functional Assessment of Cancer Therapy-Immune Checkpoint Modulator (FACT-ICM).^21^^,^^22^ However, these instruments vary considerably in their developmental background, scope of application, symptom dimensions, and measurement quality. Furthermore, most have not been systematically validated across different contexts, and no universally recognized “gold standard” instrument currently exists.^22^ This situation poses a significant challenge for instrument selection in both clinical settings and research endeavors, compromising the accuracy of symptom assessment and the comparability of study results. To date, no systematic review has evaluated the psychometric properties of PROMs for symptom assessment in the cancer immunotherapy population. This deficiency not only limits a systematic judgment of the quality of existing instruments but also hinders the advancement of scientific, standardized instrument selection and clinical practice.

Assessing the measurement properties of PROMs necessitates a standardized and rigorous methodology. To this end, the Consensus-based Standards for the selection of Health Measurement Instruments (COSMIN) initiative developed widely adopted guidelines for systematically reviewing the quality of PROMs.^23^ However, the COSMIN has noted that existing systematic reviews of PROMs often have limitations in key areas, including search strategies, how bias is assessed, how measurement properties are evaluated, and how evidence is synthesized.^24^ Addressing these issues and improving the scientific rigor and reproducibility of instrument evaluation, COSMIN released Version 2.0 of its Guideline for Systematic Reviews of PROMs and accompanying tools in 2024.^24^ These methodological updates provide a more rigorous, systematic, and transparent framework, significantly enhancing the quality appraisal of PROMs measurement property studies.

This study will systematically review the methodological quality and measurement properties of currently available PROMs for symptom assessment in patients undergoing cancer immunotherapy, adhering strictly to the COSMIN 2.0 guidelines.^24^ We intend to provide evidence-based insights to health care professionals and investigators, guiding them in the selection of high-quality, context-appropriate instruments. Furthermore, this review endeavors to influence the future design of more precise symptom assessment tools and to guide the subsequent validation of existing instruments.

## Methods

We conducted and reported this systematic review in accordance with the PRISMA (Preferred Reporting of Items for Systematic reviews and Meta-Analyses)-COSMIN (Consensus-based Standards for the selection of health Measurement Instruments) for Outcome Measurement Instruments (OMIs) 2024 guideline.^25^ The protocol was registered prospectively with PROSPERO (Registration No. CRD420250655464).

### Search strategy

For the identification of all pertinent studies, a systematic search strategy was constructed, encompassing four core concepts: “cancer,” “immunotherapy,” “PROMs,” and “measurement properties.” The concepts for “cancer” and “immunotherapy” were searched using controlled vocabulary (e.g., MeSH) and free-text keywords. The concepts for “PROMs” and “measurement properties” were searched using highly sensitive filters,^26^^,^^27^ the latter being the modified version by Elsman et al.^28^

We systematically searched PubMed, Scopus, Cochrane Library, Web of Science, Embase, CINAHL, CNKI, Vip, WanFang, and SinoMed database from their inception to February 10, 2025. For databases where the direct application of filters was not feasible, we employed a keyword strategy that mirrored the core logic of the filters to ensure conceptual consistency. Additionally, we manually searched the reference sections of all included articles as part of an ancestry search. The detailed search strategy for each database is provided in Appendix A.

### Inclusion and exclusion criteria

We included studies that satisfied the following criteria: (1) the study population consisted of adult patients with cancer receiving immunotherapy; (2) the research involved evaluating the measurement properties of PROMs for symptoms related to cancer immunotherapy; (3) the instrument was a self-report questionnaire or scale; and (4) The article was issued in English or Chinese, and its full text was retrievable.

Exclusion criteria were applied as follows: (1) the PROM was used solely as an outcome measure without evaluation of its properties; (2) the PROM served only as a reference standard to validate another instrument; (3) the publication type was a review, conference abstract, commentary, or editorial; (4) repeatedly published literature.

### Literature screening

All retrieved citations were imported into EndNote X9 for duplicate removal. The literature screening was conducted independently by two researchers, both of whom were trained in evidence-based methodology. The selection of studies was based on the predefined eligibility criteria. The literature screening was performed in two phases. Initially, titles and abstracts were reviewed to identify potentially relevant studies. Subsequently, the full texts of the articles that passed the initial screening were assessed for eligibility. We documented the specific reasons for rejecting any article at the full-text review stage. To resolve discrepancies, a third researcher was consulted to arbitrate the disagreements between the two initial reviewers.

### Data extraction

Data from the final included studies was independently extracted by two reviewers using a pre-designed data collection form. The extracted information was organized into two parts: Study Characteristics (Table 1): This included author (year), PROM, country, PROM language, study design, Sample size and participants, and cancer types. PROM Characteristics (Table 2): This included the PROM and reference, theoretical framework, adherence to guidelines, target population, intended context of use, administration method, number of subscales and items, response options, range of scores, scoring algorithm, recall period, and completion time.

**Table 1:** Study characteristics.

Author (year)	PROM	Country	PROM language	Study design	Sample size and participants	Cancer type
Hansen et al.^21^ (2019)	FACT-ICM	Canada	English	Cross-sectional study	37 patients with a median (range) age of 60.00 (24.00–85.00)	Endocrine (5.00%), gastrointestinal (8.00%), genitourinary (8.00%), gynecology (11.00%), head and neck (5.00%), lung (3.00%), melanoma (49.00%), sarcoma (11.00%)
Meng et al.^22^ (2022)	FACT-ICM-C	China	Chinese	Cross-sectional study	354 patients with with a mean age of 59.26 ​± ​10.68 years	Lung(36.44%), esophageal (9.04%), gastric/gastroesophageal junction (32.49%), intestinal (5.37%), gynecologic (1.41%), urinary system (2.54%), hepatobiliary pancreatic (4.24%), head and neck (5.65%), others (2.82%)
Wang et al.^20^ (2022)	MDASI-CAR	America	English	Longitudinal study	78 patients with with a mean age of 58.82 ​± ​14.28 years	Diffused large B-cell lymphoma (87.18%), follicular lymphoma (6.41%), acute lymphoblastic leukaemia (3.85%), mantle cell lymphoma (2.56%)
Mendoza et al.^18^(2020)	MDASI-immunotherapy EPT	America	English	Longitudinal study	145 patients with a mean age of 57.00 ​± ​12.90 years	Colorectal (9.00%), skin (non-melanoma) (9.00%), ovarian (8.00%), cervix (7.00%), sarcoma (6.00%), others (61.00%)
Wu et al.^19^ (2023)	MDASI-immunotherapy EPT-C	China	Chinese	Cross-sectional study	312 patients with a mean age of 47.03 ​± ​13.05 years	Colorectal
He et al.^34^(2023)	SAS-ICI	China	Chinese	Cross-sectional study	229 patients with a mean age of 53.53 ​± ​15.61 years	Lung (51.50%), lymphoma (48.50%)
Fan et al.^33^ (2024)	SRSI-irAEs-LC	China	Chinese	Cross-sectional study	512 patients with a mean age of 61.73 ​± ​9.56 years	Lung
Yan et al.^35^(2023)	SSS-CIP	China	Chinese	Cross-sectional study	222 patients with a mean age of 61.36 ​± ​9.89 years	Lung (21.60%), gastric (13.50%), esophageal (13.50%), others (51.40%)

PROM, Patient-reported outcome measurements; MDASI-Immunotherapy EPT, MD Anderson Symptom Inventory for early-phase (immunotherapy) trials; MDASI-Immunotherapy EPT-C, Chinese version of MDASI-Immunotherapy EPT; MDASI-CAR, MD Anderson Symptom Inventory for chimeric antigen receptor T-cell therapy; FACT-ICM, Functional Assessment of Cancer Therapy-Immune Checkpoint Modulator; FACT-ICM-C, Chinese version of FACT-ICM; SRSI-irAEs-LC, Self-Report Symptom Inventory of immune-related Adverse Events in Patients with Lung Cancer; SAS-ICI, Symptom Assessment Scale for Tumor Immune Checkpoint Inhibitor Therapy; SSS-CIP, Symptom Self-report Scale for Cancer Immunotherapy Patients.

**Table 2:** Patient-reported outcome measure characteristics.

PROM and reference	Theoretical framework	Adherence to guidelines	Target population	Intended context of use	Administration method	Number of subscales and items	Response options	Range of scores	Scoring algorithm	Recall period	Completion time
FACT-ICM, Hansen et al.^21^ (2019)	None	FDA	Cancer patients aged 18 or older Undergoing ICIs treatment	Clinical practice and clinical trials	Self-report or interview	52 items with 2 subscales: Functional assessment of cancer therapy-general and immune checkpoint modulators	5-Point Likert scale	0–208	The subscale totals are summed	7 d	10–15 minutes
FACT-ICM-C, Meng et al.^22^ (2022)	None	FDA	Cancer patients aged 18 or older Undergoing ICIs treatment	Clinical practice	Self-report or interview	52 items with 2 subscales: Functional assessment of cancer therapy-general and immune checkpoint modulators	5-Point Likert scale	0–208	The subscale totals are summed	7 d	_
MDASI-CAR, Wang et al.^20^ (2022)	None	FDA	Patients aged 18 and above with hematologic malignancies undergoing chimeric antigen receptor T-cell therapy	Clinical trials	Self-report or interview	29 items with 2 subscales: Chimeric antigen receptor T-cell Moduleand and MDASI interference	11-Point Likert scale	0–290	The subscale totals are summed	24 h	Within 5 minutes
MDASI-immunotherapy EPT, Mendoza et al.^18^ (2020)	None	None	Cancer patients aged 18 or older Undergoing Immunotherapy	Early-phase clinical trials	Self-report or interview	26 items with 2 subscales: Early-phase trials (immunotherapy) module and MDASI interference	11-Point Likert scale	0–260	The subscale totals are summed	24 h	Within 5 minutes
MDASI-immunotherapy EPT-C, Wu et al.^19^ (2023)	None	None	Cancer patients aged 18 or older Undergoing Immunotherapy	Clinical practice and clinical trials	Self-report or interview	26 items with 2 subscales: Early-phase trials (immunotherapy) module and MDASI interference	11-Point Likert scale	0–260	The subscale totals are summed	24 h	_
SAS-ICI, He et al.^34^ (2023)	None	None	Cancer patients aged 18 or older Undergoing ICIs treatment	Clinical practice	Self-report	18 items with 4 dimensions: Skin symptoms, Nervous/digestive symptoms, Skeletal symptoms, and Respiratory symptoms	5-Point Likert scale	0–60	The subscale totals are summed	14 d	Within 10 minutes
SRSI-irAEs-LC, Fan et al.^33^ (2024)	Theory of Unpleasant symptoms	FDA	Lung cancer patients aged 18 and above undergoing programmed cell death protein 1/programmed cell death ligand 1 ICIs treatment.	Clinical trials	Self-report	26 items with 8 dimensions: Skin symptoms, Digestive system symptoms, Respiratory system symptoms, Bone and muscle symptoms, Neurological system symptoms, Eyes symptoms, Cardiac symptoms, and general symptoms	5-Point Likert scale	0–130	The subscale totals are summed	21 d	11–19 minutes
SSS-CIP, Yan et al.^35^ (2024)	Theory of Unpleasant symptoms	FDA	Cancer patients aged 18 or older Undergoing Immunotherapy	Clinical practice	Self-report	48 items with 2 subscales: Symptom Occurrence and symptom impact	1 to 5 rating scale	48–240	The subscale totals are summed	With in one treatm–ent cycle	14.34 ​± ​1.33 minutes

PROM, Patient-reported outcome measurements; FACT-ICM, Functional Assessment of Cancer Therapy-Immune Checkpoint Modulator; FACT-ICM-C, Chinese version of FACT-ICM; MDASI-CAR, MD Anderson Symptom Inventory for chimeric antigen receptor T-cell therapy; MDASI-Immunotherapy EPT, MD Anderson Symptom Inventory for early-phase (immunotherapy) ​trials; MDASI-Immunotherapy EPT-C, Chinese version of MDASI-Immunotherapy EPT; SAS-ICI, Symptom Assessment Scale for Tumor Immune Checkpoint Inhibitor Therapy; SRSI-irAEs-LC, Self-Report Symptom Inventory of immune-related Adverse Events in Patients with Lung Cancer; SSS-CIP, Symptom Self-report Scale for Cancer Immunotherapy Patients; MDASI, MD Anderson Symptom Inventory; FDA, Food and Drug Administration; ICIs, immune checkpoint inhibitors; “–” ​= ​not report in the study.

### Quality assessment

For the quality assessment, two researchers worked independently and utilized the COSMIN User Manual for Systematic Reviews of PROMs Version 2.0 as their guide.^29^ This process utilized the COSMIN Risk of Bias checklist version 3.0 and the COSMIN criteria for good measurement properties version 2.0.^30^^,^^31^ A third researcher was engaged to resolve any discrepancies through discussion, ensuring the consistency and reliability of the final ratings.

#### Assessment of methodological quality

An evaluation of the risk of bias within the included studies was performed based on the criteria from the COSMIN Risk of Bias checklist version 3.0.^30^ This checklist evaluates 10 domains: PROM development, content validity, structural validity, internal consistency, cross-cultural validity/measurement invariance, reliability, measurement error, criterion validity, hypotheses testing for construct validity, and responsiveness. Each standard within the domains was rated as “Very Good,” “Adequate,” “Doubtful,” or “Inadequate.” Since methodological weaknesses in one area cannot be offset by strengths in another, the “worst-score-counts” principle was utilized to establish the overall quality rating for each measurement property per study.

#### Assessment of measurement properties

The COSMIN criteria for good measurement properties version 2.0 provided the framework for evaluating the measurement properties of the PROMs, an analysis that included all 10 domains mentioned previously.^31^ For each property, the corresponding evidence was assigned a rating of “Sufficient” (+), “Insufficient” (−), or “Indeterminate” (?). The results were first rated per study and then qualitatively synthesized across studies for the same PROM. If results were consistent, the overall rating mirrored the single-study rating. An exploration of potential heterogeneity sources (e.g., differing populations or variations in study quality) was undertaken in response to any inconsistencies in the findings. If heterogeneity could be explained, results were summarized separately for subgroups or exclusively for high-quality studies. If unexplained, the overall rating was determined by either: (1) rating the result as inconsistent (±), or (2) basing the rating on the majority of results, provided that at least 75% of the studies showed consistent findings.

#### Assessment of evidence quality

The overall evidence for each measurement property was assigned a quality grade based on a modified Grading of Recommendations Assessment, Development and Evaluation (GRADE) system.^32^ Two reviewers rated the evidence as “High,” “Moderate,” “Low,” or “Very Low” based on four factors: risk of bias, inconsistency, imprecision, and indirectness. The initial quality grading for the evidence was set to “High”. This rating was subsequently downgraded upon the identification of these factors. For internal consistency, the initial evidence level was determined by the evidence for structural validity. The GRADE system was not applied to properties with an overall “indeterminate” result.^24^ A third reviewer resolved any disagreements.

#### Formulation of recommendations

Based on the COSMIN 2.0 guidelines,^24^ the evaluated PROMs were assigned to one of three categories of recommendation: Category A (Recommended for use): PROMs with evidence of sufficient measurement properties for all domains, supported by high-quality evidence. Category B (Not recommended for use): PROMs with evidence of at least one insufficient measurement property, supported by high-quality evidence. Category C (Cannot yet be recommended): All other PROMs.

If multiple Category A tools existed, they would be further compared on their measurement quality, feasibility, and interpretability to select the optimal instrument. If all tools were in Category B, the most promising instrument based on content validity would be identified, and recommendations for its improvement would be proposed. If all tools were in Category C, priority would be given to instruments with at least low-quality evidence of sufficient content validity, and an agenda for further validation research would be formulated.^24^

## Results

### Literature search

**Fig. 1:**
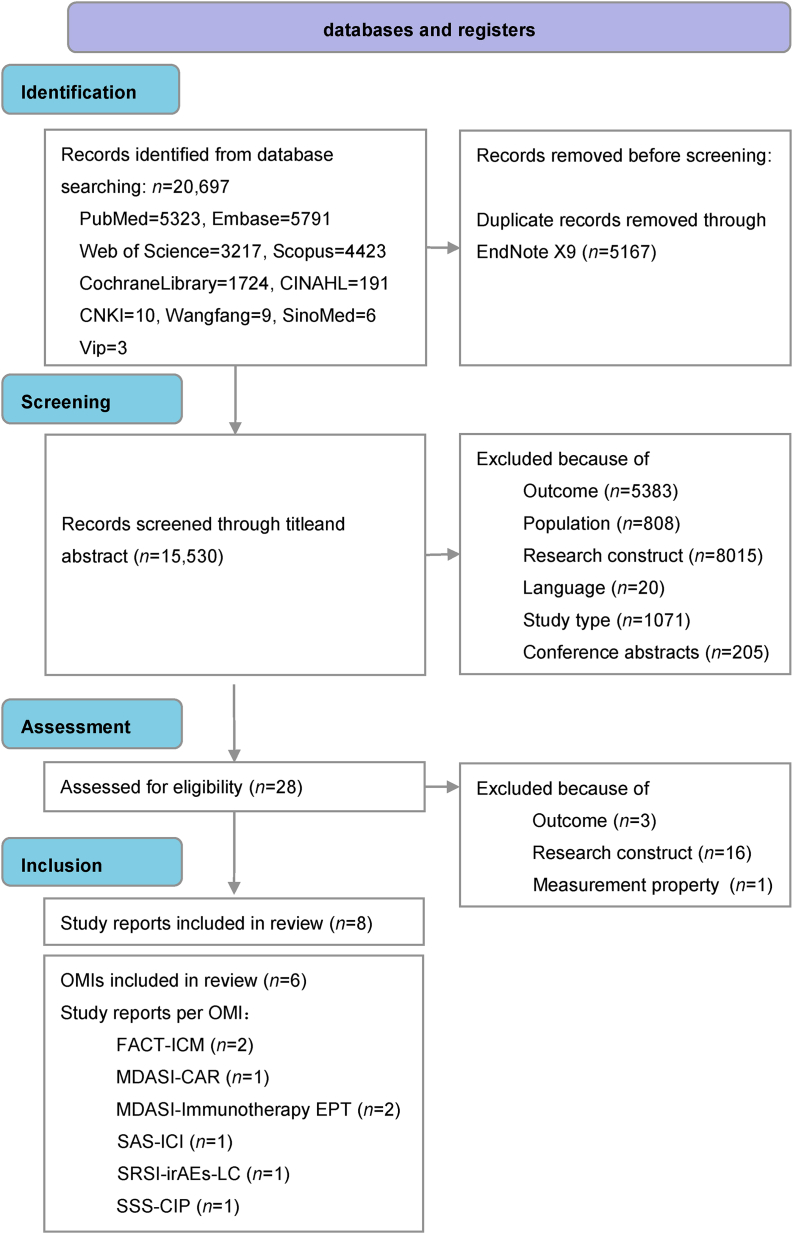
PRISMA-COSMIN flow diagram. PRISMA, Preferred Reporting of Items for Systematic reviews and Meta-Analyses; COSMIN, Consensus-based Standards for the selection of health Measurement Instruments; OMIs, Outcome Measurement Instruments; FACT-ICM, Functional Assessment of Cancer Therapy-Immune Checkpoint Modulator; MDASI-CAR, MD Anderson Symptom Inventory for chimeric antigen receptor T-cell therapy; MDASI-Immunotherapy EPT, MD Anderson Symptom Inventory for early-phase (immunotherapy) trials; SAS-ICI, Symptom Assessment Scale for Tumor Immune Checkpoint Inhibitor Therapy; SRSI-irAEs-LC, Self-Report Symptom Inventory of immune-related Adverse Events in Patients with Lung Cancer; SSS-CIP, Symptom Self-report Scale for Cancer Immunotherapy Patients.

As shown in Fig. 1, the systematic search of the 10 databases initially identified 20,697 articles. Following a rigorous screening process and full-text review, 8 articles met the inclusion criteria.^18^, ^19^, ^20^, ^21^, ^22^^,^^33^, ^34^, ^35^ From these studies, we identified a total of 6 distinct PROMs, which were then subjected to data extraction and quality assessment.

### Characteristics of included studies and PROMs

As shown in Table 1, this review includes a total of 8 studies, with 6 published in English^18^, ^19^, ^20^, ^21^, ^22^^,^^33^ and two in Chinese.^34^^,^^35^ The publication dates of these studies range from 2019 to 2024. The included studies comprised a total of 1889 patients. The size of these individual study populations ranged from 37 to 512 participants. The studies were conducted in the United States, Canada, and China. The cancer types covered in these studies include lung cancer, gastrointestinal cancer, head and neck cancer, and others.The majority of the research designs were cross-sectional studies.

As shown in Table 2, the PROMs included in the review exhibit the following characteristics in terms of development, application, and structure. In terms of theoretical foundation, more than half of the scales were developed based on Food and Drug Administration (FDA) guidelines,^21^^,^^22^^,^^33^^,^^35^ while only two scales were guided by the Theory of Unpleasant Symptoms.^33^^,^^35^ Regarding the target population and application scenarios, these scales are primarily designed for broad cancer patients receiving ICIs treatment, with the goal of clinical practice application. Only two tools were designed for specific populations: the MDASI-CAR designed for patients with blood malignancies undergoing chimeric antigen receptor T-cell therapy,^20^ and the Self-Report Symptom Inventory of immune-related Adverse Events in Patients with Lung Cancer (SRSI-irAEs-LC),^33^ designed for lung cancer patients undergoing programmed cell death protein 1/programmed cell death ligand one inhibitor therapy.In terms of structure and management features, two-thirds of the scales include two subscales: “core symptoms” and “functional interference,” with a total number of items ranging from 15 to 52.^18^, ^19^, ^20^^,^^35^ Although the recall period varies from 24 hours to 21 days, most tools can be completed in less than 20 minutes, ensuring low burden for clinical application.

### Methodological quality and measurement properties of included instruments

Our assessment of the included instruments' methodological quality and measurement properties identified several notable shortcomings. Only three of the instruments reported a complete development process,^21^^,^^33^^,^^35^ the remainder lacked critical phases, such as concept elicitation or undergoing pilot testing. The scope of the psychometric evaluations also differed significantly across the studies. The number of measurement properties assessed per instrument ranged from just one to six, with only three instruments reporting on five or more properties.^22^^,^^33^^,^^34^ Furthermore, crucial properties such as cross-cultural validity/measurement invariance and measurement error were not reported for any of the included instruments.

#### Quality assessment and results for PROM development and content validity

Table 3 presents the quality assessment and ratings for the PROM development process and content validity. During the conceptual elicitation phase, four studies reported their detailed processes.^20^^,^^21^^,^^33^^,^^35^ The methodological quality for the MDASI-CAR^20^ and FACT-ICM^21^ was rated “Very Good” due to their rigorous approaches. In contrast, the quality for the SRSI-irAEs-LC^33^ and the Symptom Self-report Scale for Cancer Immunotherapy Patients (SSS–CIP)^35^ was rated “Doubtful” because the qualifications of the interviewers were not clearly reported. Despite these differences in methodological quality, all four instruments demonstrated a clear conceptual basis and achieved data saturation, leading to a “Sufficient” (+) rating for this measurement property.Four studies reported conducting pilot testing.^19^^,^^21^^,^^33^^,^^35^ The methodological quality for most of these was rated “Doubtful” because the comprehensibility of the response options was not evaluated. Notably, the Chinese version of the MDASI-Immunotherapy EPT^19^ relied solely on quantitative data for this assessment, also resulting in a “Doubtful” quality rating. However, as patients generally reported the instrument was easy to understand and complete, its property was still rated as “Sufficient” (+).

**Table 3:** Quality assessment and results for PROM development and content validity.

PROM	PROM development (Concept elicitation study)		PROM development (Pilot study)	Content validity (Patient consultation)			Content validity (Expert consultation)		
	Relevance	Comprehen-siveness	Comprehen-sibility	Relevance	Comprehen-siveness	Comprehen-sibility	Relevance	Comprehen-siveness	Comprehen-sibility
FACT-ICM^21^	VG/+	VG/+	D/?				VG/+	VG/+	VG/+
FACT-ICM-C^22^				D/+	D/+	D/+	D/+	D/+	D/+
MDASI-CAR^20^	VG/+	VG/+							
MDASI-immunotherapy EPT^18^									
MDASI-immunotherapy EPT-C^19^			D/+						
SAS-ICI^34^							D/+	D/+	
SRSI-irAEs-LC^33^		D/+	D/?				D/+	D/+	D/+
SSS-CIP^35^	D/+	D/+	D/?				D/+	D/+	

PROM, Patient-reported outcome measurements; FACT-ICM, Functional Assessment of Cancer Therapy-Immune Checkpoint Modulator; FACT-ICM-C, Chinese version of FACT-ICM; MDASI-CAR, MD Anderson Symptom Inventory for chimeric antigen receptor T-cell therapy; MDASI-Immunotherapy EPT, MD Anderson Symptom Inventory for early-phase (immunotherapy) ​trials; MDASI-Immunotherapy EPT-C, Chinese version of MDASI-Immunotherapy EPT; SAS-ICI, Symptom Assessment Scale for Tumor Immune Checkpoint Inhibitor Therapy; SRSI-irAEs-LC, Self-Report Symptom Inventory of immune-related Adverse Events in Patients with Lung Cancer; SSS-CIP, Symptom Self-report Scale for Cancer Immunotherapy Patients.

Blank cells indicate data were not reported; "/" is preceded by methodological quality and followed by psychometric properties ratings; “VG" ​= ​Very Good; “D" ​= ​Doubtful; "+" ​= ​Sufficient; "-" ​= ​Insufficient; "?" ​= ​Indeterminant.

The formal assessment of content validity, conducted in new patient populations distinct from the development phase, involved gathering patient and expert feedback on the instruments' relevance, comprehensiveness, and comprehensibility.^24^ Among the five studies reporting on content validity,^21^^,^^22^^,^^33^, ^34^, ^35^ only the FACT-ICM^21^ achieved a “Very Good” rating for methodological quality. We assigned a “Doubtful” rating to the remaining studies because they provided insufficient reporting on their research processes and statistical methods. The Chinese version of the FACT-ICM^22^ was the only study to integrate feedback from both patients and experts, achieving “Sufficient” (+) ratings for relevance, comprehensiveness, and comprehensibility from both groups. The other studies assessed content validity by consulting experts only. However, as their findings met the COSMIN criteria, their content validity from the expert perspective was also rated as “Sufficient” (+).

#### Internal structure

In the evaluation of the internal structure of the measurement instruments, only structural validity and internal consistency were assessed, as detailed in Table 4, due to a lack of information on cross-cultural validity/measurement invariance. According to the COSMIN 2.0 guidelines, factor analysis is the preferred method for evaluating structural validity, with confirmatory factor analysis (CFA) being more appropriate for instruments with a well-defined dimensional structure.^24^

**Table 4:** Methodological quality and ratings of psychometric properties.

PROM	Structural validity	Internal consistency	Reliability	Criterion validity	Hypotheses testing for construct validity	Responsiveness
FACT-ICM^21^						
FACT-ICM-C^22^	A/+	VG/+	D/+		a. A/+ b. VG/+	
MDASI-CAR^20^		VG/?			a. A/+ b. VG/+	
MDASI-immunotherapy EPT^18^		VG/+			b. VG/+	VG/?
MDASI-immunotherapy EPT-C^19^	A/+	VG/+		D/-	b. VG/+	
SAS-ICI^34^	A/+	VG/+	D/?	D/-		
SRSI-irAEs-LC^33^	VG/+	VG/+	D/-	D/-	b. VG/+	
SSS-CIP^35^	VG/-	VG/?				

PROM, Patient-reported outcome measurements; FACT-ICM, Functional Assessment of Cancer Therapy-Immune Checkpoint Modulator; FACT-ICM-C, Chinese version of FACT-ICM; MDASI-CAR, MD Anderson Symptom Inventory for chimeric antigen receptor T-cell therapy; MDASI-Immunotherapy EPT, MD Anderson Symptom Inventory for early-phase (immunotherapy) ​trials; MDASI-Immunotherapy EPT-C, Chinese version of MDASI-Immunotherapy EPT; SAS-ICI, Symptom Assessment Scale for Tumor Immune Checkpoint Inhibitor Therapy; SRSI-irAEs-LC, Self-Report Symptom Inventory of immune-related Adverse Events in Patients with Lung Cancer; SSS-CIP, Symptom Self-report Scale for Cancer Immunotherapy Patients.

Blank cells indicate data were not reported; "/" is preceded by methodological quality and followed by psychometric properties rating; “VG" ​= ​Very Good; “D" ​= ​Doubtful; "+" ​= ​Sufficient; "-" ​= ​Insufficient; "?" ​= ​Indeterminant; “A" ​= ​Adequate; “a" ​= ​Convergent validity; “b" ​= ​Known-Groups validity.

Of the five studies that assessed structural validity, three employed both CFA and exploratory factor analysis,^22^^,^^33^^,^^35^ while the remaining two used only EFA.^19^^,^^34^ The methodological quality of the latter two was rated as “adequate.” The Chinese version of the FACT-ICM^22^ was also rated as “adequate” in its methodological quality, as its sample size was five to seven times the number of items. The methodological quality of the other studies was rated as “very good.” In the structural validity assessment of the SSS-CIP,^35^ the comparative fit index (CFI) was less than 0.95, indicating “insufficient” psychometric properties. However, the other studies met the COSMIN quality criteria, demonstrating “sufficient” psychometric properties.

Regarding internal consistency, all seven studies that reported this attribute calculated Cronbach's alpha, and their methodological quality was rated as “very good.” Five of these studies provided at least low-quality evidence for structural validity and reported Cronbach's alpha values of ≥ 0.70.^18^^,^^19^^,^^22^^,^^33^^,^^34^ Therefore, their psychometric properties were rated as “sufficient.” The other two studies,^20^^,^^35^ however, did not assess structural validity, leading to an “indeterminate” rating for their psychometric properties.

#### Remaining measurement properties

The quality assessment of the remaining measurement properties is presented in Table 4.

**Reliability:** Only three studies evaluated the reliability (test-retest) of their respective instruments. A significant limitation is that none of the studies verified that patients' conditions were stable between the two measurement points. Furthermore, there was a lack of reporting on whether the administration methods and settings remained consistent. Consequently, their methodological quality was rated as “doubtful.” Among these, the Chinese version of the FACT-ICM^22^ reported an intraclass correlation coefficient (ICC) greater than 0.7, indicating “sufficient” psychometric properties. In contrast, the SRSI-irAEs-LC^33^ had an ICC of less than 0.7, which was deemed “insufficient.” The third study^34^ did not report an ICC or any other relevant correlation coefficient, resulting in an “indeterminate” rating for its psychometric properties.

**Criterion Validity:** Criterion validity was reported only for the Chinese version of the MDASI-Immunotherapy EPT,^19^ the SRSI-irAEs-LC,^33^ and the SAS-ICI.^34^ All of these studies used a non-"gold standard” instrument as the comparator, and all reported correlation coefficients below 0.7. As a result, their methodological quality was rated as “doubtful,” and their psychometric properties were rated as “insufficient."

**Hypothesis Testing for Construct Validity:** Five studies reported on hypothesis testing for construct validity,^18^, ^19^, ^20^^,^^22^^,^^33^ primarily examining convergent and known-groups validity. The MDASI-CAR^20^ and the Chinese version of the FACT-ICM^22^ reported on both convergent and known-groups validity, whereas the other scales only addressed known-groups validity. For convergent validity, the MDASI-CAR^20^ and the Chinese version of the FACT-ICM^22^ calculated Pearson correlations but failed to provide the score distributions or mean scores of the two instruments being compared. Thus, their methodological quality was rated as “adequate.” In contrast, all scales assessing known-groups validity provided detailed descriptions of subgroup characteristics and between-group differences, leading to a “very good” methodological quality rating. As all studies^18^, ^19^, ^20^^,^^22^^,^^33^ demonstrated that ≥ 75% of their results were consistent with predefined hypotheses, their psychometric properties were rated as “sufficient."

**Responsiveness:** Only the MDASI-Immunotherapy EPT^18^ assessed responsiveness. The study featured a robust design and appropriate statistical methods, earning a “very good” rating for methodological quality. However, because it did not provide definitive results on responsiveness, its psychometric properties were rated as “indeterminate."

### Summary of measurement properties and evidence quality for included instruments

This study provides a synthesis evaluation of the measurement properties of the same instrument based on the COSMIN User Manual version 2.0.^29^ Table 5 presents the synthesized results and the corresponding quality of evidence, which ranged from “high” to “very low.” The overall results indicate that only the FACT-ICM^21^^,^^22^ demonstrated sufficient results supported by high-quality evidence for content validity, structural validity, and internal consistency. The other instruments exhibited varying levels of psychometric performance and evidence quality.

**Table 5:** Synthetic results and quality of evidence for the psychometric properties of assessment tools were included.

PROM	Content validity			Structural validity	Internal consistency	Reliability	Criterion validity	Hypotheses testing for construct validity	Responsiveness
	Relevance	Comprehen-siveness	Comprehen-sibility						
FACT-ICM^21^^,^^22^	+/High	+/High	+/High	+/High	+/High	+/Low		a. +/Moderate b. +/High	
MDASI-CAR^20^	+/Moderate	+/Moderate			?/NA			a. +/Low b. +/Moderate	
MDASI-immunotherapy EPT^18^^,^^19^			+/Low	+/Moderate	+/Moderate		-/Low	b. +/Moderate	?/NA
SAS-ICI^34^	+/Low	+/Low		+/Moderate	+/Moderate	?/NA	-/Low		
SRSI-irAEs-LC^33^	+/Low	+/Low	±/Low	+/High	+/High	-/Low	-/Low	b. +/Moderate	
SSS-CIP^35^	+/Very low	+/Very low	+/Low	-/Moderate	?/NA				

PROM, Patient-reported outcome measurements; FACT-ICM, Functional Assessment of Cancer Therapy-Immune Checkpoint Modulator; MDASI-CAR, MD Anderson Symptom Inventory for chimeric antigen receptor T-cell therapy; MDASI-Immunotherapy EPT, MD Anderson Symptom Inventory for early-phase (immunotherapy) ​trials; SAS-ICI, Symptom Assessment Scale for Tumor Immune Checkpoint Inhibitor Therapy; SRSI-irAEs-LC, Self-Report Symptom Inventory of immune-related Adverse Events in Patients with Lung Cancer; SSS-CIP, Symptom Self-report Scale for Cancer Immunotherapy Patients.

Blank cells indicate data were not reported; "/" is preceded by overall rating and followed by the quality of evidence; NA=Not Applicable; “+” ​= ​Sufficient; “-” ​= ​Insufficient; "±" ​= ​inconsistent; "?" ​= ​Indeterminant; “a" ​= ​Convergent validity; “b" ​= ​Known-Groups validity.

The decision to downgrade the quality of evidence for most instruments was driven mainly by concerns about risk of bias and imprecision. Inconsistency was a specific reason for downgrading only the SRSI-irAEs-LC.^33^ It is also important to note that the GRADE system could not be applied to the reliability of the SAS-ICI^34^ or the internal consistency of the SSS-CIP,^35^ as the results were rated “indeterminate.” The reasons for downgrading the evidence quality for each instrument are detailed in the following subsections.

#### FACT-ICM

Despite its generally strong performance, the FACT-ICM's^21^^,^^22^ evidence quality was also subject to downgrades, primarily concerning risk of bias and imprecision. For risk of bias, hypothesis testing for construct validity (convergent validity) was downgraded by one level due to “adequate” methodological quality. For imprecision, reliability (test-retest) was downgraded by two levels due to a sample size of less than 50 participants.

#### MDASI-CAR

The reasons for the downgrade of MDASI-CAR^20^ are similar to those for FACT-ICM,^21^^,^^22^ both attributable to risk of bias and imprecision. Regarding risk of bias, the relevance and comprehensiveness aspects of content validity were each downgraded by one level, as evidence was based on a single concept elicitation study of “very good” quality, with no corresponding content validity study reported. Furthermore, hypothesis testing for construct validity (convergent validity) was downgraded by one level due to “adequate” methodological quality. For imprecision, hypothesis testing (both convergent and known-groups validity) was downgraded by one level due to insufficient sample size (< 100 participants).

#### MDASI-immunotherapy EPT

The evidence quality for MDASI-Immunotherapy EPT^18^^,^^19^ was primarily downgraded due to risk of bias. Specifically, content validity (comprehensibility) and criterion validity were downgraded by two levels because of “doubtful” methodological quality. Structural validity and hypothesis testing for construct validity (convergent validity) were downgraded by one level due to “adequate” methodological quality.

#### SAS-ICI

The reasons for the downgrade of SAS-ICI^34^ also primarily stem from the risk of bias. In particular, content validity (relevance and comprehensiveness) and criterion validity were both downgraded by two levels due to “doubtful” methodological quality. Structural validity was downgraded by one level due to “adequate” methodological quality.

#### SRSI-irAEs-LC

The evidence quality for SRSI-irAEs-LC^33^ was downgraded due to risk of bias, inconsistency, and imprecision. For risk of bias, content validity (relevance and comprehensiveness) and criterion validity were downgraded by two levels due to “doubtful” methodological quality. Content validity (comprehensibility) was downgraded by one level, also due to the “doubtful” methodological quality of the pilot and content validity studies. For inconsistency, content validity (comprehensibility) was downgraded by one level because of conflicting results between the expert consultation and the pilot study. For imprecision, reliability (test-retest) was downgraded by two levels due to a sample size of less than 50 participants.

#### SSS-CIP

The evidence quality for SSS-CIP^35^ was mainly downgraded due to risk of bias. Content validity (relevance and comprehensiveness) was downgraded by three levels because the concept elicitation study was of “doubtful” quality. Additionally, content validity (comprehensibility) was downgraded by two levels as the pilot study was of “doubtful” quality, and structural validity received a one-level downgrade due to “adequate” methodological quality.

### Recommendations for instrument selection

This systematic review concluded that no “gold standard” currently exists among PROMs for symptom assessment in cancer immunotherapy. Consequently, criterion validity was not considered when formulating recommendations.^24^ For all other measurement properties, no single instrument was supported by high-quality evidence to be rated as"sufficient”, nor was any property found to be definitively"insufficient”. Therefore, all included instruments were classified as Category C, indicating they cannot be recommended for use at this time.

However, following the guidance from the COSMIN User Manual version 2.0,^29^ when all available instruments are classified as Category C, the most promising instrument should be identified. In light of this, the review identifies the FACT-ICM^21^^,^^22^ as the most promising option. This is based on high-quality evidence demonstrating its sufficient content validity, structural validity, and internal consistency.Despite this, clinicians and nurse researchers considering the FACT-ICM^21^^,^^22^ must proceed with caution. Further validation is urgently needed for its unreported measurement properties, particularly responsiveness and measurement error. Furthermore, it is crucial to recognize that the FACT-ICM^21^^,^^22^ is designed to assess the impact of symptoms on a patient's quality of life, rather than to directly quantify the severity of the symptoms themselves. This distinction is critical for accurate clinical interpretation and future research applications.

## Discussion

### Main findings

This systematic review, drawing from 10 databases and yielding 8 eligible articles, evaluated 6 PROMs for symptom assessment in cancer immunotherapy. Adhering to the COSMIN 2.0 guidelines,^24^ we analyzed their methodological quality, measurement properties, and the overall quality of evidence. Our findings indicate that no one instrument is supported by an evidence base sufficient to warrant a “highly recommended” rating. Beyond revealing the prevailing limitations of existing evidence, this review highlights the coexistence of divergent measurement strategies, thereby offering clinicians a more profound conceptual framework for instrument selection. We contend that this finding also provides a critical perspective for future research agendas and policy development.

One of the most critical findings of this review is the identification of two distinct measurement strategies coexisting within the available instruments. On one hand, instruments like the MDASI series (MDASI-Immunotherapy EPT, MDASI-CAR)^18^, ^19^, ^20^ feature a clear structural separation between two modules: symptom severity and symptom interference with function. This delineation, which treats “symptom intensity” and “symptom impact” as related but distinct constructs, represents a more lucid design that allows clinicians and researchers to independently capture these different dimensions of the patient experience. In contrast, instruments such as the FACT-ICM^21^^,^^22^ tend to conflate the symptom itself with the subjective distress and quality of life impact it causes within their items (e.g., “I have joint pain,” “My rash has been bothering me”). This “fused” approach measures a more composite construct: the “overall symptom burden”. Our review, through the lens of the COSMIN framework, illuminates this dichotomy and, crucially, underscores the potential mismatch between instrument choice and assessment goals that may arise. This finding highlights an urgent need for a well-validated, high-quality instrument focused specifically on quantifying symptom severity itself.

In addition to this conceptual divergence, we identified widespread methodological deficiencies, which largely explain the low quality of evidence. Content validity is a cornerstone of any measurement instrument,^28^ yet most tools reviewed showed significant flaws in its assessment. According to COSMIN standards,^24^ high-quality content validity assessment requires systematic patient input during development, followed by validation in an independent patient cohort. However, we found most studies to be inadequate: 1) Neglecting patient centrality: Item generation often over-relied on expert opinion or literature, failing to sufficiently capture patients' authentic and diverse experiences. While expert knowledge is valuable, it cannot fully substitute for the varied perspectives of patients. 2) Non-standardized assessment procedures: Some studies equated preliminary findings from the development phase with final evidence of content validity, omitting independent validation in a new target population. This is inappropriate as the generalizability of the tool to diverse patient groups must be confirmed. 3) Lack of critical information: Many studies failed to report the qualifications of interviewers or to explain the rationale behind the chosen response options and recall periods. These omissions can compromise data accuracy and reliability. To address these issues, future research must adhere to the COSMIN 2.0 guidelines^24^ for a comprehensive assessment of content validity and enhance interviewer training to improve data quality.

Reliability, the consistency of measurement results upon repeate.^24^^,^^36^ Only three studies reported on reliability,^22^^,^^33^^,^^34^ and most failed to provide sufficient evidence confirming that patients' clinical status remained stable during the measurement interval. This lack of attention to patient stability renders the test-retest reliability results unconvincing. Future studies should meticulously describe any changes in the clinical context between assessments, for instance, by collecting clinical data to verify patient stability.

Furthermore, a notable gap was the widespread lack of reporting on cross-cultural validity, measurement error, and responsiveness. Cross-cultural validity, which assesses whether a translated or culturally adapted instrument reflects the performance of the original version,^24^ is critical for an instrument's global applicability but was rarely evaluated. Although two studies^19^^,^^22^ involved translation of scales, they did not perform cross-cultural validation. The neglect of measurement error means we cannot determine the precision of these instruments.^37^ Additionally, the absence of responsiveness data leaves us unable to know if these tools are sensitive enough to detect clinically meaningful changes resulting from immunotherapy, thereby limiting their utility in practice. Future research should prioritize the assessment of these properties in accordance with COSMIN standards to enhance the scientific rigor and generalizability of these tools.

Based on our in-depth analysis of the measurement constructs, we recommend that instrument selection be strictly aligned with the clinical assessment goal. If the objective is to assess the “overall symptom burden"—a composite measure integrating symptom intensity and its impact on quality of life—then the FACT-ICM^21^^,^^22^ is the most promising instrument currently available. Its item design aligns with this “fused” construct, and high-quality evidence supports its sufficiency in content validity, structural validity, and internal consistency. However, clinicians and nurse researchers must recognize that it is not designed to precisely measure pure symptom severity, and evidence for its reliability, measurement error, and responsiveness remains incomplete. Conversely, if the objective is to “precisely and independently quantify the severity of specific symptoms,” a clear evidence gap exists. Although the MDASI series offers an ideal conceptual framework for this goal by separating “severity” and “interference” modules,^18^, ^19^, ^20^ its comprehensive psychometric evidence, particularly in the immunotherapy context, is insufficient. Therefore, we cannot currently recommend any existing instrument for this specific purpose.

The evidence limitations revealed by this review delineate a clear, dual-track agenda for future research. First, it is imperative to consolidate the FACT-ICM^21^^,^^22^ as a benchmark instrument for assessing “overall symptom burden” by conducting robust studies to validate its remaining measurement properties. Second, and constituting a critical research priority, is the need to develop or further validate a high-quality PROMs focused on “symptom severity.” This effort should begin with a systematic validation of existing high-potential instruments (e.g., the MDASI series). If these tools are found to be inadequate after rigorous evaluation, the development of an entirely new, patient-centric instrument should be initiated. All future validation and development efforts must adhere strictly to the COSMIN guidelines to ensure a comprehensive, high-quality assessment of all measurement properties.

### Implications for nursing practice and research

The results of this review provide clear guidance for nursing practice. When conducting symptom assessments for cancer patients receiving immunotherapy, frontline clinical nurses must first define the goal of the assessment. When the goal is to understand the combined impact of symptoms on a patient's quality of life, the FACT-ICM serves as the most promising tool for effectively assessing a patient's overall symptom burden and helping nurses understand how individual, seemingly mild symptoms can seriously impact a patient's daily life when superimposed. However, nurses should also be aware of its limitations, namely that the tool cannot accurately differentiate or track the severity of a particular symptom. And when the goal of the assessment is symptom severity, caregivers must recognize that no single tool is yet adequately supported by evidence and recommended.

At the same time, the aforementioned challenges in clinical practice point the way to future research. Given the obvious shortcomings in quantifying the severity of specific symptoms, the central task of nursing researchers is to respond to this urgent clinical need. This dictates clear research pathways: one, a comprehensive validation of the psychometric properties of potentially available tools in strict adherence to the COSMIN 2.0 guidelines;^24^ and two, if validation indicates that the existing tools are not applicable, the development of novel assessment tools specifically targeting immunotherapy-related symptoms needs to be initiated. In summary, clear assessment goals are a bridge between clinical practice and scientific research, guiding clinical nurses in the judicious selection and application of tools and driving researchers to conduct rigorous tool validation and development efforts to improve the quality of care and advance the discipline.

### Limitations

Several limitations of this study warrant consideration. First, the scope of our literature search was restricted to publications in English and Chinese. This may have introduced a language bias, leading to the potential omission of relevant studies published in other languages and thus affecting the comprehensiveness of the evidence synthesized. Future systematic reviews could benefit from including a broader range of languages to ensure a more complete integration of evidence.Second, our review was highly specific in its focus, exclusively evaluating PROMs or their subscales designed to measure immunotherapy-related symptoms. Consequently, the scope of this review did not extend to comprehensive instruments primarily intended to measure broader domains such as functional status, mental health, or overall quality of life. While this focused approach was necessary for an in-depth analysis of symptom assessment tools, it also means that our conclusions do not encompass the full spectrum of immunotherapy's impact on patients' overall well-being. Future systematic reviews with a broader scope are warranted to provide a more holistic picture of the patient-reported outcome landscape in this population.Third, significant clinical heterogeneity existed across the included primary studies, particularly concerning cancer types and immunotherapy regimens (e.g., ICI monotherapy versus combination therapy, CAR-T cell therapy). This heterogeneity limits the generalizability of our findings. Therefore, our results should be interpreted as a macro-level summary of the available evidence, and their application to specific clinical settings requires caution. Future quantitative syntheses, such as meta-analyses, should employ pre-specified subgroup analyses to formally investigate the impact of this heterogeneity.

## Conclusions

This systematic review found that the literature on patient-reported outcome instruments for cancer immunotherapy symptoms is dominated by studies focused on initial tool development, with a scarcity of instruments that have been extensively validated or have reached a mature version. Consequently, no single instrument for assessing patient-reported symptoms in this context can be universally and strongly recommended, owing to insufficient high-quality evidence for their measurement properties.Therefore, we cautiously recommend the FACT-ICM as the most promising instrument currently available for the specific purpose of assessing “overall symptom burden.” However, for the distinct goal of quantifying pure “symptom severity,” no tool is sufficiently supported by evidence to be recommended.The future research agenda must therefore be dedicated to completing the evidence chain for existing high-potential instruments and, critically, to developing or validating new tools capable of precisely assessing symptom severity to address this unambiguous need in research and clinical practice.

## CRediT authorship contribution statement

**Defa Zhang**: Conceptualization, Data Curation, Visualization, Methodology, Formal analysis, Writing – Original Draft. **Yali Wang**: Conceptualization, Data Curation, Methodology, Writing – Original Draft. **Qian Wang**: Methodology, Data Curation, Supervision, Writing – Review & Editing. **Huiqing Mao**: Data Curation, Formal analysis, Visualization, Supervision. **Miaomiao Zhang**: Methodology, Software, Visualization, Validation. **Ping Xu**: Methodology, Software, Visualization, Validation. **Shuo Guo**: Data Curation, Validation, Visualization. **Rong Yan**: Project Administration, Resources, Supervision, Writing – Review & Editing. All authors have read and approved the final manuscript.

## Ethics statement

Not required.

## Data availability statement

The data that support the findings of this study are available within the article or its supplementary materials.

## Declaration of generative AI and AI-assisted technologies in the writing process

No AI tools/services were used during the preparation of this work.

## Funding

This study received no external funding.

## Declaration of competing interest

The authors declare no conflict of interest.
