# Supplementary material for: Psychometric properties of patient-reported outcome measures for symptom assessment in patients with cancer receiving immunotherapy: A systematic review following the COSMIN 2.0 guidelines
Source: Asia Pac J Oncol Nurs. 2025 Aug 19;12:100774. doi: 10.1016/j.apjon.2025.100774 (PMC12409797; doi:10.1016/j.apjon.2025.100774)
Supplement: Multimedia component 1 [file mmc1.docx]

Appendix I. Search strategy

We conducted the first search on February 10, 2025, and performed an updated search on June 20, 2025, which yielded no new eligible studies.

**PubMed search June 20, 2025**

**#1 Neoplasms**

("Neoplasms"[Mesh]) OR (tumor*[Title/Abstract] OR neoplasia[Title/Abstract] OR neoplasm[Title/Abstract] OR cancer*[Title/Abstract] OR malignancy[Title/Abstract] OR malignancies[Title/Abstract] OR carcinoma[Title/Abstract])

**#2 Immunotherapy**

(("Immunotherapy"[Mesh]) OR ("Immune Checkpoint Inhibitors"[Mesh])) OR (PD-1 Inhibitors[Title/Abstract] OR PD-L1 Inhibitors[Title/Abstract] OR CTLA-4 Inhibitors[Title/Abstract] OR CAR-T[Title/Abstract] OR Programmed Cell Death Protein 1 Inhibitors[Title/Abstract] OR Programmed Death Ligand 1 Inhibitors[Title/Abstract] OR Cytotoxic T Lymphocyte Associated Protein 4 Inhibitors[Title/Abstract] OR chimeric antigen receptor T[Title/Abstract])

**#3 PROM filter (developed by the University of Oxford, see [www.comin.nl](http://www.comin.nl/))**

"Patient Reported Outcome Measures"[Mesh] OR "Quality of Life"[Mesh] OR prom[tiab] OR proms[tiab] OR pro[tiab] OR pros[tiab] OR HRQL[tiab] OR HRQoL[tiab] OR QL[tiab] OR QoL[tiab] OR quality of life[tiab] OR life quality[tiab] OR health index*[tiab] OR health indices[tiab] OR health profile*[tiab] OR health status[tw] OR ((patient[tiab] OR self[tiab] OR child[tiab] OR parent[tiab] OR carer[tiab] OR proxy[tiab]) AND ((report[tiab] OR reported[tiab] OR reporting[tiab]) OR (rated[tiab] OR rating[tiab] OR ratings[tiab]) OR based[tiab] OR (assessed[tiab] OR assessment[tiab] OR assessments[tiab]))) OR ((disability[tiab] OR function[tiab] OR functional[tiab] OR functions[tiab] OR subjective[tiab] OR utility[tiab] OR utilities[tiab] OR wellbeing[tiab] OR well being[tiab]) AND (outcome[tiab] OR outcomes[tiab] OR index[tiab] OR indices[tiab] OR instrument[tiab] OR instruments[tiab] OR measure[tiab] OR measures[tiab] OR questionnaire[tiab] OR questionnaires[tiab] OR profile[tiab] OR profiles[tiab] OR scale[tiab] OR scales[tiab] OR score[tiab] OR scores[tiab] OR status[tiab] OR survey[tiab] OR surveys[tiab]))

**#4 Modified filter for studies on measurement properties***

((~~instrumentation[sh] OR methods[sh] OR~~ "Validation Study"[pt] ~~OR "Comparative Study"[pt]~~ OR "psychometrics"[MeSH] OR psychometr*[tiab] OR clinimetr*[tw] OR clinometr*[tw] OR "outcome assessment, health care"[MeSH] OR "outcome assessment"[tiab] OR "outcome measure*"[tw] OR "observer variation"[MeSH] OR "observer variation"[tiab] ~~OR "Health Status Indicators"[Mesh]~~ OR "reproducibility of results"[MeSH] OR reproducib*[tiab] OR "discriminant analysis"[MeSH] OR reliab*[tiab] OR unreliab*[tiab] OR valid*[tiab] OR "coefficient of variation"[tiab] ~~OR coefficient[tiab]~~ OR homogeneity[tiab] OR homogeneous[tiab] OR "internal consistency"[tiab] OR (cronbach*[tiab] AND (alpha[tiab] OR alphas[tiab])) OR (item[tiab] AND (correlation*[tiab] OR selection*[tiab] OR reduction*[tiab])) OR agreement[tw] OR precision[tw] OR imprecision[tw] OR "precise values"[tw] OR test-retest[tiab] OR (test[tiab] AND retest[tiab]) OR (reliab*[tiab] AND (test[tiab] OR retest[tiab])) OR stability[tiab] OR interrater[tiab] OR inter-rater[tiab] OR intrarater[tiab] OR intra-rater[tiab] OR intertester[tiab] OR inter-tester[tiab] OR intratester[tiab] OR intra-tester[tiab] OR interobserver[tiab] OR inter-observer[tiab] OR intraobserver[tiab] OR intra-observer[tiab] OR intertechnician[tiab] OR inter-technician[tiab] OR intratechnician[tiab] OR intra-technician[tiab] OR interexaminer[tiab] OR inter-examiner[tiab] OR intraexaminer[tiab] OR intra-examiner[tiab] OR interassay[tiab] OR inter-assay[tiab] OR intraassay[tiab] OR intra-assay[tiab] OR interindividual[tiab] OR inter-individual[tiab] OR intraindividual[tiab] OR intra-individual[tiab] OR interparticipant[tiab] OR inter-participant[tiab] OR intraparticipant[tiab] OR intra-participant[tiab] OR kappa[tiab] OR kappa 's[tiab] OR kappas[tiab] OR repeatab*[tw] OR ((replicab*[tw] OR repeated[tw]) AND (measure[tw] OR measures[tw] OR findings[tw] ~~OR result[tw] OR results[tw]~~ OR test[tw] OR tests[tw])) OR generaliza*[tiab] OR generalisa*[tiab] OR concordance[tiab] OR (intraclass[tiab] AND correlation*[tiab]) OR discriminative[tiab] OR "known group"[tiab] OR "factor analysis"[tiab] OR "factor analyses"[tiab] OR "factor structure"[tiab] OR "factor structures"[tiab] ~~OR dimension*[tiab]~~ OR subscale*[tiab] OR (multitrait[tiab] AND scaling[tiab] AND (analysis[tiab] OR analyses[tiab])) OR "item discriminant"[tiab] OR "interscale correlation*"[tiab] OR error[tiab] OR errors[tiab] OR "individual variability"[tiab] OR "interval variability"[tiab] OR "rate variability"[tiab] OR (variability[tiab] AND (analysis[tiab] OR values[tiab])) ~~OR (uncertainty[tiab] AND (measurement[tiab] OR measuring[tiab]))~~ OR "standard error of measurement"[tiab] ~~OR sensitiv*[tiab]~~ OR responsive*[tiab] OR (limit[tiab] AND detection[tiab]) OR "minimal detectable concentration"[tiab] OR interpretab*[tiab] OR ((minimal[tiab] OR minimally[tiab] OR clinical[tiab] OR clinically[tiab]) AND (important[tiab] ~~OR significant[tiab]~~ OR detectable[tiab]) AND (change[tiab] OR difference[tiab])) OR (small*[tiab] AND (real[tiab] OR detectable[tiab]) AND (change[tiab] OR difference[tiab])) OR "meaningful change"[tiab] OR "ceiling effect"[tiab] OR "floor effect"[tiab] OR "Item response model"[tiab] OR IRT[tiab] OR Rasch[tiab] OR "Differential item functioning"[tiab] OR DIF[tiab] OR "computer adaptive testing"[tiab] OR "item bank"[tiab] OR "cross-cultural equivalence"[tiab]))

**#5**

**（**#1 AND #2 AND #3 AND #4）NOT (( 'delphi-technique '[ti] OR cross-sectional[ti] OR "address"[Publication Type] OR "biography"[Publication Type] OR "case reports"[Publication Type] OR "comment"[Publication Type] OR "directory"[Publication Type] OR "editorial"[Publication Type] OR "festschrift"[Publication Type] OR "interview"[Publication Type] OR "lecture"[Publication Type] OR "legal case"[Publication Type] OR "legislation"[Publication Type] OR "letter"[Publication Type] OR "news"[Publication Type] OR "newspaper article"[Publication Type] OR "patient education handout"[Publication Type] OR "popular work"[Publication Type] OR "congress"[Publication Type] OR "consensus development conference"[Publication Type] OR "consensus development conference, nih"[Publication Type] OR "practice guideline"[Publication Type]) NOT ("animals"[MeSH Terms] NOT "humans"[MeSH Terms]))

**Embase search June 20, 2025**

**#1 Neoplasms**

'neoplasm'/exp OR tumor*:ab,ti OR neoplasia:ab,ti OR neoplasm:ab,ti OR cancer*:ab,ti OR malignancy:ab,ti OR malignancies:ab,ti OR carcinoma:ab,ti

**#2 Immunotherapy**

'immunotherapy'/exp OR 'immune checkpoint inhibitor'/exp OR 'pd-1 inhibitors':ab,ti OR 'pd-l1 inhibitors':ab,ti OR 'ctla-4 inhibitors':ab,ti OR 'car t':ab,ti OR 'programmed cell death protein 1 inhibitors':ab,ti OR 'programmed death ligand 1 inhibitors':ab,ti OR 'cytotoxic t lymphocyte associated protein 4 inhibitors':ab,ti OR 'chimeric antigen receptor t':ab,ti

**#3 PROM filter (developed by the University of Oxford, see [www.comin.nl](http://www.comin.nl/))**

'hr-pro':ab,ti OR 'hrpro':ab,ti OR 'hrql':ab,ti OR 'hrqol':ab,ti OR 'ql':ab,ti OR 'qol':ab,ti OR 'quality of life':ab,ti OR 'life quality':ab,ti OR 'health index':ab,ti OR 'health indices':ab,ti OR 'health profile':ab,ti OR 'health profiles':ab,ti OR 'health status':ab,ti OR ('patient':ab,ti OR 'self':ab,ti OR 'child':ab,ti OR 'parent':ab,ti OR 'carer':ab,ti OR 'proxy':ab,ti AND ('report':ab,ti OR 'reported':ab,ti OR 'reporting':ab,ti OR 'rated':ab,ti OR 'rating':ab,ti OR 'ratings':ab,ti OR 'based':ab,ti OR 'assessed':ab,ti OR 'assessment':ab,ti OR 'assessments':ab,ti)) OR ('disability':ab,ti OR 'function':ab,ti OR 'functional':ab,ti OR 'functions':ab,ti OR 'subjective':ab,ti OR 'utility':ab,ti OR 'utilities':ab,ti OR 'wellbeing':ab,ti OR 'well being':ab,ti AND ('outcome':ab,ti OR 'outcomes':ab,ti OR 'index':ab,ti OR 'indices':ab,ti OR 'instrument':ab,ti OR 'instruments':ab,ti OR 'measure':ab,ti OR 'measures':ab,ti OR 'questionnaire':ab,ti OR 'questionnaires':ab,ti OR 'profile':ab,ti OR 'profiles':ab,ti OR 'scale':ab,ti OR 'scales':ab,ti OR 'score':ab,ti OR 'scores':ab,ti OR 'status':ab,ti OR 'survey':ab,ti OR 'surveys':ab,ti))

**#4 Modified filter for studies on measurement properties***

~~'intermethod comparison '/exp OR~~ 'data collection method '/exp OR 'validation study '/exp OR 'feasibility study '/exp OR 'pilot study '/exp OR 'psychometry '/exp OR 'reproducibility '/exp OR reproducib*:ab,ti OR 'audit ':ab,ti OR psychometr*:ab,ti OR clinimetr*:ab,ti OR clinometr*:ab,ti OR 'observer variation '/exp OR 'observer variation ':ab,ti OR 'discriminant analysis '/exp OR 'validity '/exp OR reliab*:ab,ti OR valid*:ab,ti  ~~OR 'coefficient ':ab,ti~~  OR 'internal consistency ':ab,ti OR (cronbach*:ab,ti AND ( 'alpha ':ab,ti OR 'alphas ':ab,ti)) OR 'item correlation ':ab,ti OR 'item correlations ':ab,ti OR 'item selection ':ab,ti OR 'item selections ':ab,ti OR 'item reduction ':ab,ti OR 'item reductions ':ab,ti OR 'agreement ':ab,ti OR 'precision ':ab,ti OR 'imprecision ':ab,ti OR 'precise values ':ab,ti OR 'test-retest ':ab,ti OR ( 'test ':ab,ti AND 'retest ':ab,ti) OR (reliab*:ab,ti AND ( 'test ':ab,ti OR 'retest ':ab,ti)) OR 'stability ':ab,ti OR 'interrater ':ab,ti OR 'inter-rater ':ab,ti OR 'intrarater ':ab,ti OR 'intra-rater ':ab,ti OR 'intertester ':ab,ti OR 'inter-tester ':ab,ti OR 'intratester ':ab,ti OR 'intratester ':ab,ti OR 'interobeserver ':ab,ti OR 'inter-observer ':ab,ti OR 'intraobserver ':ab,ti OR 'intraobserver ':ab,ti OR 'intertechnician ':ab,ti OR 'inter-technician ':ab,ti OR 'intratechnician ':ab,ti OR 'intratechnician ':ab,ti OR 'interexaminer ':ab,ti OR 'inter-examiner ':ab,ti OR 'intraexaminer ':ab,ti OR 'intraexaminer ':ab,ti OR 'interassay ':ab,ti OR 'inter-assay ':ab,ti OR 'intraassay ':ab,ti OR 'intra-assay ':ab,ti OR 'interindividual ':ab,ti OR 'inter-individual ':ab,ti OR 'intraindividual ':ab,ti OR 'intra-individual ':ab,ti OR 'interparticipant ':ab,ti OR 'inter-participant ':ab,ti OR 'intraparticipant ':ab,ti OR 'intraparticipant ':ab,ti OR 'kappa ':ab,ti OR 'kappas ':ab,ti OR 'coefficient of variation ':ab,ti OR repeatab*:ab,ti OR (replicab*:ab,ti OR 'repeated ':ab,ti AND ( 'measure ':ab,ti OR 'measures ':ab,ti OR 'findings ':ab,ti ~~OR 'result ':ab,ti OR 'results ':ab,ti~~ OR 'test ':ab,ti OR 'tests ':ab,ti)) OR generaliza*:ab,ti OR generalisa*:ab,ti OR 'concordance ':ab,ti OR ( 'intraclass ':ab,ti AND correlation*:ab,ti) OR 'discriminative ':ab,ti OR 'known group ':ab,ti OR 'factor analysis ':ab,ti OR 'factor analyses ':ab,ti OR 'factor structure ':ab,ti OR 'factor structures ':ab,ti OR 'dimensionality ':ab,ti OR subscale*:ab,ti OR 'multitrait scaling analysis ':ab,ti OR 'multitrait scaling analyses ':ab,ti OR 'item discriminant ':ab,ti OR 'interscale correlation ':ab,ti OR 'interscale correlations ':ab,ti OR ( 'error ':ab,ti OR 'errors ':ab,ti AND(measure*:ab,ti OR correlat*:ab,ti OR evaluat*:ab,ti OR 'accuracy ':ab,ti OR 'accurate ':ab,ti OR 'precision ':ab,ti OR 'mean ':ab,ti)) OR 'individual variability ':ab,ti OR 'interval variability ':ab,ti OR 'rate variability ':ab,ti OR 'variability analysis ':ab,ti OR ( 'uncertainty ':ab,ti AND ( 'measurement ':ab,ti OR

'measuring ':ab,ti)) OR 'standard error of measurement ':ab,ti ~~OR sensitiv*:ab,ti~~ OR responsive*:ab,ti OR ( 'limit ':ab,ti AND 'detection ':ab,ti) OR 'minimal detectable concentration ':ab,ti OR interpretab*:ab,ti OR (small*:ab,ti AND ( 'real ':ab,ti OR 'detectable ':ab,ti) AND ( 'change ':ab,ti OR 'difference ':ab,ti)) OR 'meaningful change ':ab,ti OR 'minimal important change ':ab,ti OR 'minimal important difference ':ab,ti OR 'minimally important change ':ab,ti OR 'minimally important difference ':ab,ti OR 'minimal detectable change ':ab,ti OR 'minimal detectable difference ':ab,ti OR 'minimally detectable change ':ab,ti OR 'minimally detectable difference ':ab,ti OR 'minimal real change ':ab,ti OR 'minimal real difference ':ab,ti OR 'minimally real change ':ab,ti OR 'minimally real difference ':ab,ti OR 'ceiling effect ':ab,ti OR 'floor effect ':ab,ti OR 'item response model ':ab,ti OR 'irt ':ab,ti OR 'rasch ':ab,ti OR 'differential item functioning ':ab,ti OR 'dif ':ab,ti OR 'computer adaptive testing ':ab,ti OR 'item bank ':ab,ti OR 'cross-cultural equivalence ':ab,ti

**#5 Publicatie types**

#1 AND #2 AND #3 AND #4 AND ('article'/it OR 'article in press'/it OR 'data papers'/it)

**#6 Not animals**

#5 NOT（'animal'/exp NOT 'human'/exp）

**Web of Science search June 20, 2025**

**#1 Neoplasms**

TS=(neoplasm OR tumor* OR neoplasia OR neoplasm OR cancer* OR malignancy OR malignancies OR carcinoma)

**#2 Immunotherapy**

TS=(Immunotherapy OR Immune Checkpoint Inhibitors OR PD-1 Inhibitors OR PD-L1 Inhibitors OR CTLA-4 Inhibitors OR CAR-T OR Programmed Cell Death Protein 1 Inhibitors OR Programmed Death Ligand 1 Inhibitors OR Cytotoxic T Lymphocyte Associated Protein 4 Inhibitors OR chimeric antigen receptor T)

**#3 PROM**

TS=(Patient Reported Outcome Measures OR prom OR proms OR pro OR pros OR HRQL OR HRQoL OR QoL OR quality of life OR instrument* OR questionnaire OR scale OR index OR tool)

**#4 Measurement properties**

TS=(psychometric* OR valid* OR relia* OR measurement error OR internal consistency OR responsiveness)

**#5**

（#1 AND #2 AND #3 AND #4） NOT (TS=((address OR biography OR case reports OR comment OR directory OR editorial OR festschrift OR interview OR lecture OR legal case OR legislation OR letter OR news OR newspaper article OR patient education handout OR popular work OR congress OR consensus development conference OR consensus development conference, NIH OR practice guideline)))

**Scopus search June 20, 2025**

**#1 Neoplasms**

TITLE-ABS-KEY ( neoplasm OR tumor* OR neoplasia OR neoplasm OR cancer* OR malignancy OR malignancies OR carcinoma )

**#2 Immunotherapy**

TITLE-ABS-KEY ( immunotherapy OR "Immune Checkpoint Inhibitors" OR "PD-1 Inhibitors" OR "PD-L1 Inhibitors" OR "CTLA-4 Inhibitors" OR "CAR-T" OR "Programmed Cell Death Protein 1 Inhibitors" OR "Programmed Death Ligand 1 Inhibitors" OR "Cytotoxic T Lymphocyte Associated Protein 4 Inhibitors" OR "chimeric antigen receptor T" )

**#3 PROM**

TITLE-ABS-KEY ( "Patient Reported Outcome Measures" OR prom OR proms OR pro OR pros OR hrql OR hrqol OR qol OR "quality of life" OR instrument* OR questionnaire OR scale OR index OR tool )

**#4 Measurement properties**

TITLE-ABS-KEY ( psychometric* OR valid* OR relia* OR "measurement error" OR "internal consistency" OR responsiveness )

**#5**

（#1 AND #2 AND #3 AND #4）AND NOT ( TITLE-ABS-KEY ( address OR biography OR "case reports" OR comment OR directory OR editorial OR festschrift OR interview OR lecture OR "legal case" OR legislation OR letter OR news OR "newspaper article" OR "patient education handout" OR "popular work" OR congress OR "consensus development conference" OR "consensus development conference, NIH" OR "practice guideline" ) )

**Cochrane library search June 20, 2025**

**#1 Neoplasms**

(MeSH descriptor: [Neoplasms] explode all trees) OR ((tumor* OR neoplasia OR neoplasm OR cancer* OR malignancy OR malignancies OR carcinoma):ti,ab,kw)

**#2 Immunotherapy**

(MeSH descriptor: [Immunotherapy] explode all trees) OR

(MeSH descriptor: [Immune Checkpoint Inhibitors] explode all trees) OR ("PD-1 Inhibitors" OR "PD-L1 Inhibitors" OR "CTLA-4 Inhibitors" OR "CAR-T" OR "Programmed Cell Death Protein 1 Inhibitors" OR "Programmed Death Ligand 1 Inhibitors" OR "Cytotoxic T Lymphocyte Associated Protein 4 Inhibitors" OR "chimeric antigen receptor T"):ti,ab,kw)

**#3 PROM filter (developed by the University of Oxford, see [www.comin.nl](http://www.comin.nl/))**

HR-PRO OR HRPRO OR HRQL OR HRQoL OR QL OR QoL OR 'quality of life' OR 'life quality' OR 'health index' OR 'health indices' OR health profile* OR 'health status' OR ((patient OR self OR child OR parent OR carer OR proxy) AND ((report OR reported OR reporting) OR (rated OR rating OR ratings) OR based OR (assessed OR assessment OR assessments))) OR ((disability OR function OR functional OR functions OR subjective OR utility OR utilities OR wellbeing OR well being) AND (outcome OR outcomes OR index OR indices OR instrument OR instruments OR measure OR measures OR questionnaire OR questionnaires OR profile OR profiles OR scale OR scales OR score OR scores OR status OR survey OR surveys))

**#4 Measurement properties**

(MeSH descriptor: [Psychometrics] explode all trees) OR ((valid* OR relia* OR "measurement error" OR "internal consistency" OR responsiveness):ti,ab,kw)

**#5**

（#1 AND #2 AND #3 AND #4） NOT ( address OR biography OR "case reports" OR comment OR directory OR editorial OR festschrift OR interview OR lecture OR "legal case" OR legislation OR letter OR news OR "newspaper article" OR "patient education handout" OR "popular work" OR congress OR "consensus development conference" OR "consensus development conference, NIH" OR "practice guideline" )

**CINAHL search June 20, 2025**

**#1 Neoplasms**

SU=(neoplasm OR tumor* OR neoplasia OR neoplasm OR cancer* OR malignancy OR malignancies OR carcinoma)

**#2 Immunotherapy**

SU=(immunotherapy OR "Immune Checkpoint Inhibitors" OR "PD-1 Inhibitors" OR "PD-L1 Inhibitors" OR "CTLA-4 Inhibitors" OR "CAR-T" OR "Programmed Cell Death Protein 1 Inhibitors" OR "Programmed Death Ligand 1 Inhibitors" OR "Cytotoxic T Lymphocyte Associated Protein 4 Inhibitors" OR "chimeric antigen receptor T")

**#3 PROM**

SU=("Patient Reported Outcome Measures" OR prom OR proms OR pro OR pros OR

OR hrql OR hrqol OR qol OR "quality of life" OR instrument* OR questionnaire OR scale OR index OR tool)

**#4 Measurement properties**

SU=(psychometric* OR valid* OR relia* OR "measurement error" OR "internal consistency" OR responsiveness)

**#5**

（#1 AND #2 AND #3 AND #4） NOT ( address OR biography OR "case reports" OR comment OR directory OR editorial OR festschrift OR interview OR lecture OR "legal case" OR legislation OR letter OR news OR "newspaper article" OR "patient education handout" OR "popular work" OR congress OR "consensus development conference" OR "consensus development conference, NIH" OR "practice guideline" )

**CNKI search June 20, 2025**

主题=(癌症 + 肿瘤) AND 主题=(免疫治疗 + 免疫检查点抑制剂 +程序性死亡蛋白 + 程序性死亡受体 + PD-1 + PD-L1 + 细胞毒性T淋巴细胞抗原4 + CTLA-4 + 嵌合抗原受体T细胞 + CAR-T) AND主题=(患者报告结局 + 健康相关生活质量 + 生活质量 + 问卷 + 工具 + 量表 + 指数) AND主题=(心理测量学 + 测量误差 + 信度 + 效度 + 内部一致性 + 反应度)

**Wangfang search June 20, 2025**

题名或关键词=(癌症 + 肿瘤) AND 题名或关键词=(免疫治疗 + 免疫检查点抑制剂 +程序性死亡蛋白 + 程序性死亡受体 + PD-1 + PD-L1 + 细胞毒性T淋巴细胞抗原4 + CTLA-4 + 嵌合抗原受体T细胞 + CAR-T) AND题名或关键词=(患者报告结局 + 健康相关生活质量 + 生活质量 + 问卷 + 工具 + 量表 + 指数) AND题名或关键词=(心理测量学 + 测量误差 + 信度 + 效度 + 内部一致性 + 反应度)

**SinoMed search June 20, 2025**

( "癌症"[常用字段:智能] OR "肿瘤"[常用字段:智能]) AND( "免疫治疗"[常用字段:智能] OR "免疫检查点抑制剂 OR程序性死亡蛋白"[常用字段:智能] OR "程序性死亡受体"[常用字段:智能] OR "PD-1"[常用字段:智能] OR "PD-L1"[常用字段:智能] OR "细胞毒性T淋巴细胞抗原4"[常用字段:智能] OR "CTLA-4"[常用字段:智能] OR "嵌合抗原受体T细胞"[常用字段:智能] OR "CAR"[常用字段:智能]) AND( "患者报告结局"[常用字段:智能] OR " 健康相关生活质量"[常用字段:智能] OR "生活质量"[常用字段:智能] OR "问卷"[常用字段:智能] OR "工具"[常用字段:智能] OR "量表"[常用字段:智能] OR "指数"[常用字段:智能]) AND( "心理测量学"[常用字段:智能] OR "测量误差"[常用字段:智能] OR "信度"[常用字段:智能] OR "效度"[常用字段:智能] OR "内部一致性"[常用字段:智能] OR "反应度"[常用字段:智能])

**Vip search June 20, 2025**

题名或关键词=(癌症 + 肿瘤) AND 题名或关键词=(免疫治疗 + 免疫检查点抑制剂 +程序性死亡蛋白 + 程序性死亡受体 + PD-1 + PD-L1 + 细胞毒性T淋巴细胞抗原4 + CTLA-4 + 嵌合抗原受体T细胞 + CAR-T) AND题名或关键词=(患者报告结局 + 健康相关生活质量 + 生活质量 + 问卷 + 工具 + 量表 + 指数) AND题名或关键词=(心理测量学 + 测量误差 + 信度 + 效度 + 内部一致性 + 反应度)

*****The Terwee measurement property filter revised by Elsman et al.^28^ The crossed-out search terms were discarded because we found that when these terms were combined with the topics of this study (cancer, immunotherapy, and PROM), they retrieved a large number of irrelevant articles, significantly increasing the screening burden.

28. Elsman EBM, Mokkink LB, Langendoen-Gort M, et al. Systematic review on the measurement properties of diabetes-specific patient-reported outcome measures (PROMs) for measuring physical functioning in people with type 2 diabetes. BMJ Open Diab Res Care. 2022;10(3):e002729. https://doi.org/10.1136/bmjdrc-2021-002729.
